# A Qualitative Study of the Impact of COVID-19 on Hospice and Home-Based Palliative Care in Taiwan

**DOI:** 10.1093/geroni/igab046.3650

**Published:** 2021-12-17

**Authors:** YuHsuan (Olivia) Wang, Susan Enguidanos

**Affiliations:** 1 USC, Los Angeles, California, United States; 2 University of Southern California, Los Angeles, California, United States

## Abstract

COVID-19 has had a tremendous impact on individuals and health care, particularly among those with serious illnesses. Although Taiwan initially reported low rates of COVID-19 (20.7 cases per million in 2020), by early 2021 the Alpha variant began spreading in Taiwan, which went into a soft lockdown from May to July. This study aimed to investigate the impact of COVID-19 on Hospice and Home-Based Palliative Care (HBPC) patients, family, and medical team member in Taiwan during different COVID-related time points. From November 2020 to May 2021, we conducted semi-structured interviews with 2 patient, 5 caregivers, and 8 medical team members in the hospice and HBPC setting to elicit their views about the impact of COVID-19 on care. Content analysis was used to guide the analysis of themes. Two researchers independently coded transcripts and met to reconcile codes. Results showed varying impact across the time points. In 2020, patients, caregivers, medical team members felt little impact of COVID-19. However, in 2021, HBPC caregivers reduced the frequency of HBPC visits to lower the household COVID-19 infection risk. Hospice and HBPC team members reported that more caregivers selected HBPC over hospice. They also observed an increase in complicated grief among family survivors following the soft lockdown. The impact of the pandemic on homecare provision increased as prevalence of COVID-19 increased, impacting care and quality of hospice and HBPC. Further research is needed to better understand the impact of the pandemic on the quality of hospice /HBPC and grief experiences.

